# Epithelial-myoepithelial carcinoma of the parotid gland: Case report

**DOI:** 10.1016/j.ijscr.2025.111914

**Published:** 2025-09-05

**Authors:** Asiye Merve Erdoğan, Funda Kuş Bozkurt

**Affiliations:** a ENT Specialist in Adana Acıbadem Hospital, ENT Clinics, Adana, Turkey; b Pathologist in Gaziantep Şehitkamil State Hospital, Gaziantep, Turkey

**Keywords:** Case report, Epithelial-myoepithelial, Parotid gland, Salivary gland tumor

## Abstract

**Introduction and importance:**

Malignant salivary gland neoplasms present a significant diagnostic challenge, requiring the integration of clinical, radiological, and pathological assessments. Epithelial-myoepithelial carcinoma (EMC) is a rare malignant neoplasm that exhibits a biphasic histopathological pattern. Although the major salivary glands are most commonly affected, EMC can also involve minor salivary glands and, in rare instances, the seromucous glands of the upper aerodigestive tract.

**Presentation of case:**

A 47-year-old male presented with a right-sided parotid mass. On physical examination, a solitary, well-defined, nodular mass, approximately 3 cm in size, was palpated in the right parotid gland, demonstrating mobility. Fine-needle aspiration biopsy indicated a cystic lesion. A right superficial parotidectomy was performed with careful preservation of the facial nerve. The tumor was managed with surgery alone, with negative surgical margins. The patient had no recurrence at the 3-year follow-up.

**Clinical discussion:**

Tumors with features such as positive surgical margins, aneuploidy, nuclear atypia, solid growth pattern, increased mitotic activity, and necrosis are indicative of more aggressive behavior in EMC. Despite being classified as low-grade tumors, EMC has been associated with local recurrence and distant metastasis. In cases exhibiting aggressive characteristics, surgery alone may be insufficient, necessitating the addition of elective neck irradiation to prevent local recurrences. Achieving tumor-negative surgical margins is considered critical for reducing the likelihood of recurrence.

**Conclusion:**

EMC is a rare salivary gland malignancy that requires careful diagnostic evaluation and management. While surgery alone may be sufficient for certain cases with negative surgical margins, further treatment such as irradiation may be warranted in aggressive cases to minimize recurrence.

## Highlights

• The standard treatment for patients with localized epithelial-myoepithelial carcinoma (EMC) involves total surgical resection with clear margins and adequate extent of resection.
• In cases where the disease exhibits positive surgical margins, aneuploidy, nuclear atypia, solid growth pattern, increased mitotic activity, and necrosis, surgery alone may be insufficient.
• Neck dissection is indicated only in the presence of clinically apparent lymphadenopathy, following complete surgical excision of the primary tumor.

## 1 Introduction

Malignant salivary gland neoplasms often present considerable diagnostic challenges, requiring meticulous integration of clinical, radiological, and pathological evaluation. Epithelial-myoepithelial carcinoma (EMC) is a rare malignant salivary gland neoplasm that exhibits a biphasic histopathological pattern [1]. The World Health Organization (WHO) defines EMC as “a malignant tumor composed of variable proportions of two cell types that typically form duct-like structures. The biphasic morphology is represented by an inner layer of duct-lining, epithelial-type cells, and an outer layer of clear, myoepithelial-type cells” [1]. The first description of EMC was provided by Donath et al. in 1972, and was subsequently included in the WHO classification in 1991 [1]. This rare entity accounts for approximately 0.5 % to 1 % of all salivary gland neoplasms, further contributing to its infrequent occurrence [1]. EMC exhibits a female predominance, with a ratio of approximately 2:1 (F:M), and affects a broad age range of individuals, from 6 to 92 years of age [1].

The major salivary glands are most commonly involved, although the condition may also affect the minor salivary glands and, in rare cases, the seromucous glands within the upper aerodigestive tract [1]. Features such as positive surgical margins, aneuploidy, nuclear atypia, solid growth patterns, high mitotic activity, and necrosis are associated with a more aggressive behavior in EMC. Although EMC is generally considered a low-grade tumor, local recurrence and distant metastasis have been documented. In cases exhibiting aggressive behavior, surgery alone may not suffice and elective neck irradiation is recommended to prevent local recurrence. Although there is no established consensus regarding the optimal treatment for EMC, it is widely agreed that achieving tumor-negative surgical margins is a crucial factor in reducing the risk of recurrence [2].

This case report has been reported in line with the SCARE checklist [3].

## 2 Case presentation

A 47-year-old male presented with a right-sided parotid mass, which he noticed 5 years ago, with a progressive increase in size over the past 2 years. The patient's medical history was non-contributory.

**Fig. 1:**
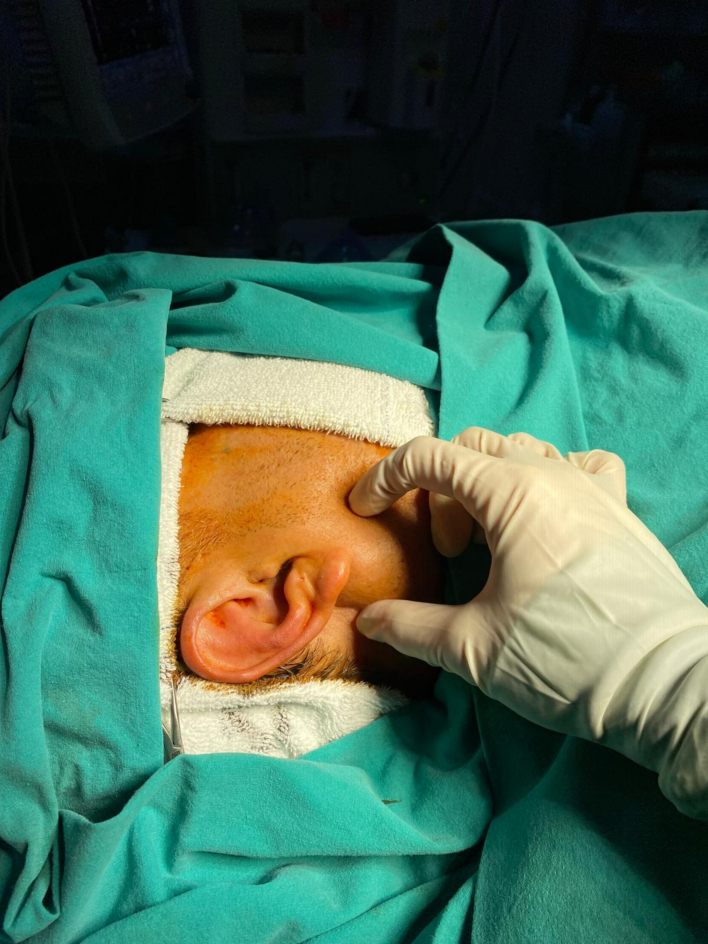
Preoperative physical examination image.

On physical examination, a solitary, well-defined, nodular mass, approximately 3 cm in size, was palpated in the right parotid gland, demonstrating mobility.(Fig.1) The overlying skin appeared unaffected, and there was no restriction of mouth opening. No cervical lymphadenopathy or facial nerve deficits were observed. Extraoral examinations did not reveal any significant alterations. Neck ultrasonography revealed a well-defined 13*10 mm lesion within the right parotid gland, containing both cystic and solid components. No lymphadenopathy was observed.

Fine-needle aspiration biopsy indicated a cystic lesion. Preoperative hematological and serological investigations were within normal limits.

**Fig. 2:**
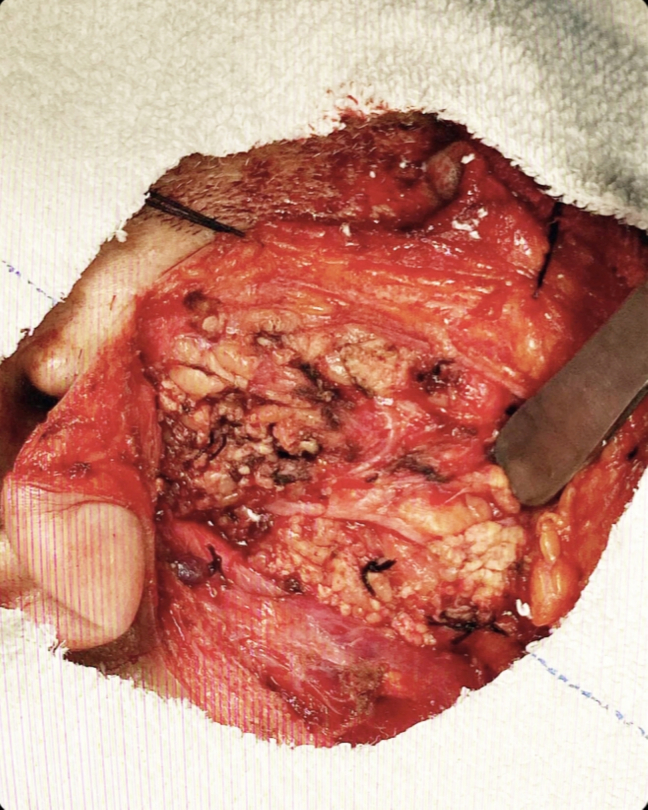
Intraoperative field following tumor excision.

**Fig. 3:**
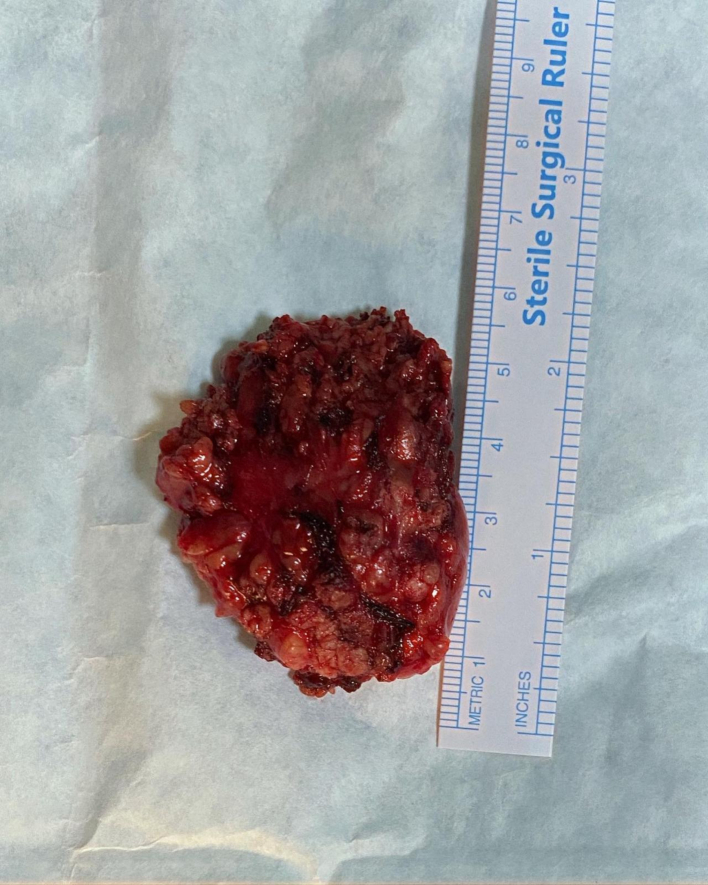
Resected tumor.

**Fig. 4- 5:**
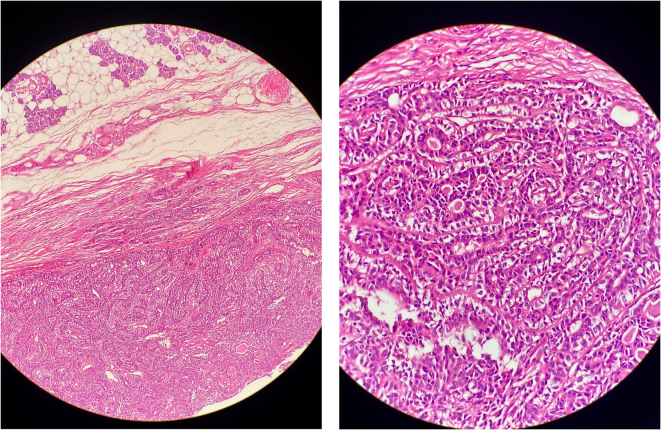
Microscopic images.

A right superficial parotidectomy was performed with careful preservation of the facial nerve without any nerve monitoring two weeks after the initial examination. (Fig.2) There were no complications or adverse outcomes. Macroscopic examination of the surgical specimen revealed a 3 × 3 × 2 cm mass. (Fig.3) Microscopic examination of the surgical specimen revealed duct-like structures lined by a single layer of epithelial cells with eosinophilic cytoplasm surrounded by a layer of clear myoepithelial cells within a hyalinized stroma. (Fig.4–5) To confirm this diagnosis, immunohistochemical studies for Ki-67, pan-cytokeratin, and p63 were conducted. The abluminal cells showed immunoreactivity for p63, whereas the luminal cells were positive for pan-cytokeratin. The Ki-67 positivity index, which can be used to predict the proliferative activity of cells, was 10 %. The final pathological diagnosis was epithelial-myoepithelial carcinoma (EMC), and no tumor involvement was detected at the surgical margins.

A PET-CT scan performed 4 months postoperatively at an external center showed no pathological findings.

No recurrence was observed during the 3-year follow-up, which included physical examinations every three months and annual ultrasound evaluations. Our case has been followed up without recurrence for a relatively long period compared to the literature.

## 3 Discussion

Epithelial-myoepithelial carcinoma (EMC) was included in the World Health Organization (WHO) classification of salivary gland neoplasms in 1991 and was assigned ICD-O-3 code 8562/3 [4]. While commonly referred to as EMC, this tumor has also been identified by other names, including tubular carcinoma, clear cell myoepithelioma, adenomyoepithelioma, and clear cell carcinoma [1,4]. This rare neoplasm exhibits a female predominance, with an average age of onset occurring in the 6th to 7th decades of life [1,4]. Approximately 60 % of EMC cases are observed in the parotid gland, although it may also affect minor salivary glands and, in rare instances, seromucous glands within the upper aerodigestive tract [1].

Due to its infrequency, only a limited number of EMC cases have been documented to date. One such case was reported by Shome et al., who described a 60-year-old male patient with EMC of the left retromolar trigone. This case arose following incisional biopsy of a 2 × 2 × 1 cm mass. However, owing to the patient's poor economic status, follow-up was not possible. This case represents a rare occurrence of EMC in an unusual anatomical location, specifically the retromolar trigone, and is likely the only reported instance of EMC in this location to date [5].

Another case, presented by Riaz et al., involved a 37-year-old male patient with stage 4a EMC in the parapharyngeal space. The tumor measured 9 × 5.5 cm. In addition to intensity-modulated radiation therapy, the patient received a regimen of 33 fractions of radiation therapy and chemotherapy, which included 100 mg/m^2^ cisplatin administered every three weeks. Researchers reported that this treatment successfully controlled the disease locally for more than eight years, with no associated toxicities, such as xerostomia, resulting from radiotherapy [2].

In a case report by Yadav et al., a 40-year-old female patient presented with a 4 × 4 cm mass in the root of the tongue and a 1 × 1 cm lymph node in the right cervical region. An incisional biopsy of the tongue mass revealed a subepithelial infiltrative tumor; however, no evidence of perineural or lymphovascular invasion, anaplasia, increased mitotic activity, or necrosis was noted. The patient exhibited bilateral lung metastasis on chest radiography, although biopsy was not performed because of the patient's refusal. This case was unusual because the EMC exhibited metastatic behavior without high-grade histological features. Given the advanced stage of the disease, the patient was referred for radiochemotherapy [6].

Waltzman et al. performed surgical resection with a 1-cm margin using endoscopy for epithelial-myoepithelial carcinoma (EMC) originating from the nasal septum and the right middle concha. Owing to the presence of local residues,radiotherapy was administered using pencil beam scanning proton therapy. No recurrence was observed during the 6-month follow-up period. Notably, this is the first reported case in which proton therapy was employed for the treatment of EMC. Given the proximity of the positive surgical margin to the skull base, pencil beam scanning proton therapy was selected as the preferred treatment modality [7].

In the case reported by Khattab et al., a 58-year-old female patient presented with a mass in the hard palate, which had been neglected for 35 years and had grown to a size of 45 mm, involving the right and left maxilla, left upper alveolar protrusion, and part of the nasal septum. Initially staged as T4N0M0 with a presumptive diagnosis of carcinoma, the diagnosis could not be confirmed via incisional biopsy. After total maxillectomy and reconstruction with a free fibula flap, at definitive diagnosis of EMC was established. The patient had no evidence of metastasis or recurrence during the 2-year follow-up period. The authors speculated that the lesion may have resulted from the malignant transformation of a primary benign palatal pleomorphic adenoma [8].

Kaur et al. staged a 4 × 3 cm EMC tumor involving the soft palate and uvula in a 56-year-old female patient, T2N0M0. The patient underwent external beam radiotherapy (EBRT) with intensity-modulated radiotherapy (IMRT) using a 6 MV Linac to deliver a total dose of 54 Gy across 30 fractions, followed by a simultaneous integrated boost (SIB) to the high-risk gross tumor volume (GTV), escalating the dose to 66 Gy. Nine weeks post-treatment, contrast-enhanced computed tomography (CECT) of the head and neck revealed a heterogeneously enhancing oropharyngeal mass with necrotic areas located in the soft palate, extending into the retromolar trigone and along the left lateral pharyngeal wall, measuring 29 mm × 25 mm × 26 mm. The scan indicated a minimal reduction in tumor size compared with the prior CECT images. A repeat biopsy of the lesion showed features consistent with the baseline biopsy findings. No positron emission tomography (PET) scans were performed post-treatment. Following radiotherapy, the patient was treated with a chemotherapy regimen comprising ifosfamide mesna, cisplatin, and etoposide. At the 12-month follow-up, after completing 6 cycles of chemotherapy, the patient achieved complete remission [9].

The management of epithelial-myoepithelial carcinomas (EMCs) originating from minor salivary glands remains poorly defined, particularly regarding the role of radiotherapy. In contrast, adjuvant radiotherapy is commonly recommended for major salivary gland tumors when the primary tumor exceeds 4 cm in size or when positive surgical margins are present [10].

In a case report by Miura et al., the findings obtained from magnetic resonance imaging (MRI) and fine needle aspiration cytology (FNAC) suggested a diagnosis of pleomorphic adenoma in the superficial lobe of the parotid gland. However, postoperative histopathological examination, along with immunostaining and genetic tests, led to a diagnosis of epithelial-myoepithelial carcinoma (EMC). Postoperative pathological evaluation revealed that a portion of the resection margin was positive for tumor cells. Despite being informed about the potential risk of recurrence, the patient declined additional treatment and a conservative watch-and-wait approach was adopted. Thirteen months postoperatively, a cutaneous metastasis was identified in the left infraaural region. The metastasis was excised under local anesthesia, and resection margins were found to be negative. The patient subsequently followed a course of treatment without further complications [11].

In their study of 15 patients with Salivary Intercalated Duct lesions, McLean et al. investigated the relationship between recurrent the β-catenin (CTNNB1) gene and HRAS (Harvey rat sarcoma virus) mutations and epithelial-myoepithelial carcinoma (EMC). They concluded that the majority of Intercalated Duct Lesions were neoplastic, and they observed molecular overlap between these lesions, EMC, and basal cell adenomas. This study support the hypothesis that Intercalated Duct Lesions may serve as precursors of these tumors. Furthermore, the authors noted that a significant proportion of cases did not exhibit oncogenic factors, suggesting that some Intercalated Duct Lesions might be reactive and hyperplastic in nature. They concluded that Intercalated Duct Lesions represent a diverse spectrum of morphological and molecular features, encompassing both hyperplastic and neoplastic lesions [12].

In a study involving two case presentations, Yasuda et al. examined the HRAS mutation as a diagnostic molecular marker and potential target for the treatment of EMC of the parotid gland. They reported that neither of the two cases exhibited the characteristic features of epithelial-myoepithelial carcinoma; however, the presence of HRAS mutations led to diagnosis. The study further indicated that HRAS mutations were present in 81.7 % of EMC cases, although such mutations were not identified in other salivary gland tumors with characteristics similar to EMC, including basal cell adenoma, basal cell adenocarcinoma, adenoid cystic carcinoma, pleomorphic adenoma, and myoepithelial carcinoma. Additionally, the authors found no significant correlation between the presence of HRAS mutations and histological indicators of tumor aggressiveness, emphasizing that HRAS mutations alone were insufficient to diagnose EMC, as they have also been observed in other subtypes of salivary gland carcinomas, including salivary duct carcinomas.

The study concluded that total surgical resection represents the standard treatment for patients with localized epithelial–myoepithelial carcinoma (EMC), with favorable prognosis generally associated with extensive resections that achieve clear surgical margins. However, there is currently no established standard of care for recurrent, unresectable, or metastatic EMC. The authors proposed that HRAS mutations may serve as valuable diagnostic and predictive biomarkers for targeted therapies—such as tipifarnib—in cases of metastatic or recurrent disease. [13].

In a study by Vaz et al. of 246 patients, patients with high-grade tumors or tumors larger than 4 cm exhibited the poorest survival rates. Although 41 % of patients received radiotherapy in addition to surgery, the treatment did not improve the 10-year survival rate [14]. According to the French Rare Head and Neck Cancer Network (REFCOR), there is no indication for adjuvant radiotherapy following complete surgical resection in stage I and II low-grade tumors such as EMC [15]. Although the effectiveness of chemotherapy remains uncertain, some authors have suggested the use of chemoradiotherapy when surgical excision margins are inadequate [10].

## 4 Conclusion

Although epithelial-myoepithelial carcinoma (EMC) is classified as a low-grade tumor, it carries a 23–50 % risk of local recurrence and a 25 % likelihood of distant metastasis [4]. Neck dissection is indicated only in the presence of clinically evident lymphadenopathy after complete surgical excision of the primary tumor [1]. The role of adjuvant chemoradiotherapy after surgery remains a topic of ongoing debate [1]. Factors such as positive surgical margins, aneuploidy, nuclear atypia, solid growth pattern, increased mitotic activity, and necrosis are associated with more aggressive tumor behavior in EMC [4]. Long-term follow-up is critical for early detection of recurrence. Although there is no consensus regarding the optimal treatment approach for EMC, it is widely considered that achieving tumor-negative surgical margins is a crucial factor in minimizing the risk of recurrence. Our case has been followed up without recurrence for a relatively long period compared to the literature.

## Patient consent

Written informed consent was obtained.

## Ethical approval

At our institution, Acıbadem Adana Hospital, ethical approval is not required for single case reports; however, written informed consent was obtained from the patient for the publication of this case report and accompanying images.

## Funding

The study sponsors had no involvement.

## Author contributions

Asiye Merve Erdoğan MD- Design, planning, literature survey, data collection, critical review.

Funda Kuş Bozkurt MD - Pathological examination and pathological images.

## Guarantor

Asiye Merve Erdoğan MD.

Funda Kuş Bozkurt MD.

## Research registration number

Not applicable

## Conflict of interest statement

The authors declare that there is no conflicts of interest.
